# Confocal microscopic and dermoscopic features of eccrine poroma: A case report

**DOI:** 10.1111/srt.13174

**Published:** 2022-05-31

**Authors:** Weiwei Wu, Chengcheng Xiong, Yan Zhang, Xianfeng Fang

**Affiliations:** ^1^ Department of Dermatology, The First College of Clinical Medical Sciences China Three Gorges University Yichang China; ^2^ Department of Dermatology Yichang Central People's Hospital Yichang China

Dear Editor,

Eccrine poroma (EP), as first described in 1956 by Goldman et al.,^1^ is a rare benign adnexal neoplasms originating from intraepidermal region of the eccrine sweat duct that mainly affects the palmoplantar skin. Clinically, the lesions usually present as pinkish, red, flesh‐coloured, dome‐shaped papules or nodules with smooth or verrucous surfaces that are easily misdiagnosed as basal cell carcinoma, pyogenic granuloma, squamous cell carcinoma and seborrheic keratosis.

Reflectance confocal microscopy (RCM), which has shown to be an important in vivo, non‐invasive diagnostic technique for skin lesions. Effectiveness of RCM for inflammatory and neoplastic skin diseases has been widely reported in literature.^2^ Nowadays, dermoscopy is an in indispensable part of the clinical skin examination and greatly improves the clinical diagnosis of many skin tumours. EP have rarely been reported to use RCM examine lesion, and there have been only four such reports in the literature, the four patients ranged in age from 41 to 74 years, and half the patients’ pathologic diagnoses were pigmented EP.^3^, ^4^, ^5^, ^6^ In order to raise awareness of this rare condition among dermatologists, we demonstrate confocal microscopic and dermoscopic features of a case of EP.

Herein, we report a 25‐year‐old Asian male presented with an asymptomatic papule on his right chest for 1 year, without history of trauma, chronic irritation or radiation at the site of the tumour. Upon cutaneous examination, the lesion was sessile, firm, reddish‐pink, well‐circumscribed and elevated at the periphery, and a small amount of bleeding can be seen on the surface (Figure 1). The patient was generally in perfect health, and the systematic examination showed no obvious abnormalities. Histopathologic examination of the lesion showed proliferation of basal epidermal cells extending into the dermis as broad anastomosing bands. The tumour composed of monomorphic cuboid cells with deeply basophilic round nuclei. These cells formed small, round or oval ducts, and no cellular atypia was observed. The stroma contained eosinophilic hyalinized collagen, and small vessels were seen under epidermis. Histopathological manifestations suggested the diagnosis of EP (Figure 2A,B). After confocal and dermoscopy examine had been performed, the lesion was excised. No recurrence or malignant metastasis occurred within a 2‐year follow‐up.

**FIGURE 1:**
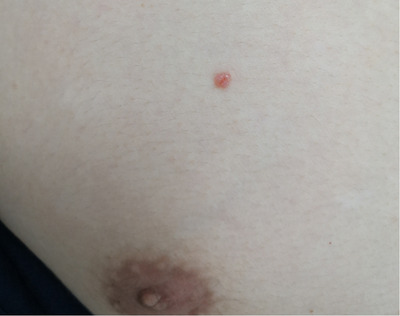
Well‐circumscribed and reddish‐pink papuloid tumour

**FIGURE 2:**
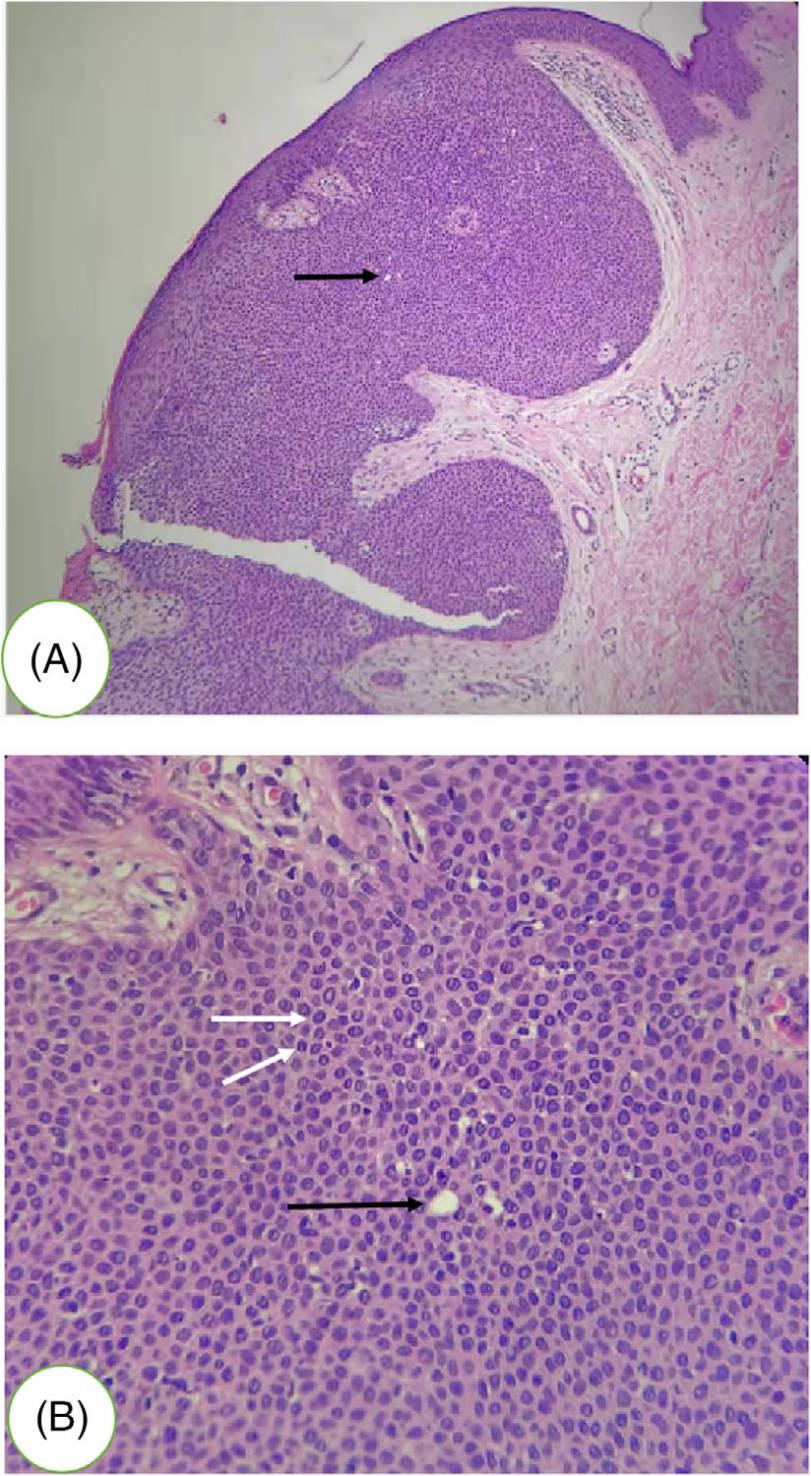
Histopathologic examination revealed broad anastomosing bands of small cuboidal basaloid cells (white arrow), extending into the dermis from the lower portion of the epidermis. Note the intracytoplasmic eccrine duct lumina (black arrow)

Confocal microscopic analysis revealed a well‐demarcated tumour consists of bright tumour islands with surrounding homogeneous dark stroma. Tumour cells were highly refractile, uniform in size and shape, round and dark nuclei and smaller than epidermal keratinocytes. Tumour cells arranged in an honeycombed structure. Hyperreflective inflammatory cells can be seen in the dermal papillary layer (Figure 3A,B). Owing to the lack of peripheral palisading of elongated cells and dark peritumoural clefts, the RCM imagings were not indicative of basal cell carcinoma.^7^ The RCM features for cuticle layer are normal, and the dermal papillary rings are integrated that proved to be a benign epithelial proliferation.

**FIGURE 3:**
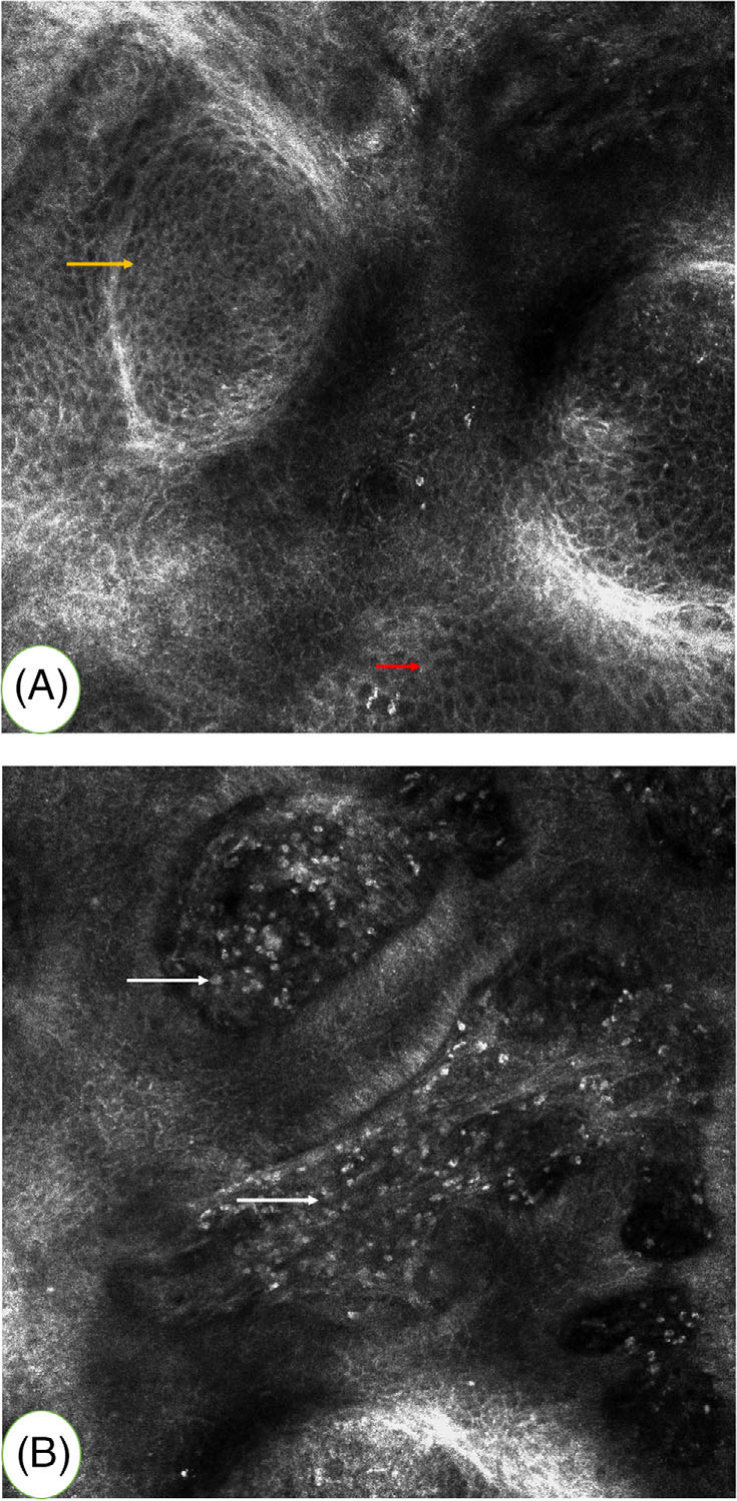
(A) Reflectance confocal microscopy (RCM) imagings showed tumour cells (yellow arrow) organized in an honeycombed pattern are brighter and smaller than surrounding epithelial cells (red arrow). (B) Hyperreflective inflammatory cells can be seen in the dermal papillary layer (white arrow)

Dermoscopy of the lesion showed linear irregular, hairpin, glomerular and poorly visualized vessels. White structureless area and perivascular light pink halos were also be observed (Figure 4). Marchetti et al. reported the most common dermoscopic features of poroma, included white interlacing areas around vessels, yellow structureless areas, milky‐red globules, poorly visualized vessels and branched vessels with rounded endings.^8^

**FIGURE 4:**
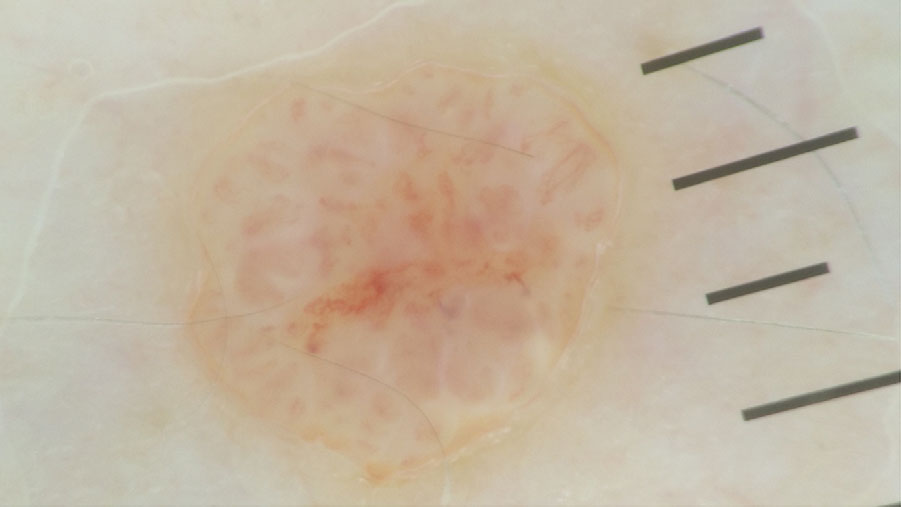
Dermoscopy presenting showed linear irregular, hairpin, glomerular and poorly visualized vessels

Our case serves as one of the few reported cases of using RCM and dermoscopy observed EP lesion and this young patient's lesion located at an unfrequent site. Given the uncommonness of the neoplasms, the diagnosis mostly relies on the histopathological examinan. RCM and dermoscopy may assist in the correct diagnosis of this neoplasm and help rule out some differential diagnoses. Due to about 18% of poromas will progress to porocarcinomas,^9^ early and correct diagnosis with auxiliary examination and histopathological examination are particularly important.

## CONFLICT OF INTEREST

The authors declare that there is no conflict of interest that could be perceived as prejudicing the impartiality of the research reported.
